# Impact of Metformin on Periodontal and Peri-Implant Soft and Hard Tissue

**DOI:** 10.3390/ijerph20021095

**Published:** 2023-01-08

**Authors:** Faisal E. Aljofi, Aminah Alesawy, Bader Alzaben, Marwa Alshaikh, Norah Alotaibi, Hajer A. Aldulaijan, Sami Alshehri, Eman Aljoghaiman, Yousif A. Al-Dulaijan, Mishali AlSharief

**Affiliations:** 1 Department of Preventive Dental Sciences, College of Dentistry, Imam Abdulrahman Bin Faisal University, P.O. Box 1982, Dammam 31441, Saudi Arabia; 2 Fellowship in Periodontics Program, College of Dentistry, Imam Abdulrahman Bin Faisal University, P.O. Box 1982, Dammam 31441, Saudi Arabia; 3 Department of Periodontics and Community Dentistry, College of Dentistry, King Saud University, Riyadh P.O. Box 60169, Saudi Arabia; 4 Department of Biomedical Dental Sciences, College of Dentistry, Imam Abdulrahman Bin Faisal University, P.O. Box 1982, Dammam 31441, Saudi Arabia; 5 Department of Substitutive Dental Sciences, College of Dentistry, Imam Abdulrahman Bin Faisal University, P.O. Box 1982, Dammam 31441, Saudi Arabia

**Keywords:** alveolar bone, diabetes, gingiva, peri-implant health, periodontal health

## Abstract

Periodontal and peri-implant soft and hard tissue in diabetic patients have always been a topic of interest for researchers and clinicians alike. Among which, a subtopic that has attracted more attention is the beneficial effect of metformin (MF) on periodontal and peri-implant soft and hard tissue. This review aimed to assess the impact of MF on the periodontal and peri-implant soft- and hard-tissue healing among diabetic patients. Research was conducted using the keywords ‘metformin’, ‘diabetes’, ‘periodontitis’, ‘implant’, and ‘peri-implantitis’ via the Medline (PubMed) and Google Scholar databases. Selected articles were reviewed. A total of 21 articles, discussing the impact on periodontal health (six animal studies, seven clinical studies, and three systematic reviews) and five studies on peri-implant health (four animal studies and one clinical study) were included. All have reported a positive impact of MF on decreasing the inflammatory response, oxidative stress, and ultimate bone loss. Similarly, human studies reported a positive effect of MF on clinical and radiographic parameters compared with controls. Despite systematic reviews reporting heterogeneity among the included studies, MF has shown a positive impact on periodontal health. In animal, clinical studies, and systematic reviews, MF showed a protective impact on periodontal and peri-implant health.

## 1. Introduction

Bone is a highly dynamic tissue that maintains levels of calcium in blood, serves as a lever for muscle action, and provides soft tissue with mechanical support [1]. Bone marrow cells can differentiate into a variety of cells, including osteoblasts, chondrocytes, and adipocytes [2]. The metabolic condition of bone marrow is determined by balancing adipogenesis and osteogenesis. This osteoblast–adipocyte ratio balance is affected by several factors, such as hormones and medications [3].

According to the American Diabetes Association, “Diabetes is a group of metabolic diseases characterized by hyperglycemia resulting from defects in insulin secretion, insulin action, or both”. Insulin resistance develops as a result of autoimmune destruction of pancreatic β-cells leading to insulin deficiency.

The literature suggests that diabetics have a substantial risk of developing periodontitis. The odds of patients with type 2 diabetes having periodontitis increase threefold compared to subjects without diabetes [4]. Over time, uncontrolled diabetic patients suffer increasing risk of bone and periodontal attachment loss [5]. Individuals diagnosed with type 2 diabetes are commonly prescribed oral hypoglycemic drugs [6].

Metformin (dimethyl biguanide) acts as a hypoglycemic by lowering the hepatic glucose production. Additionally, it is considered the first-line oral blood hypoglycemic agent for treatment of type 2 diabetes [7].

It has been proven that antidiabetic drugs such as metformin (MF) have a beneficial effect on bone tissue, affect osteoblasts, and reduce the risk of fractures among diabetic patients [8,9]. In a Danish case–control study that was conducted to evaluate the relation between MF use and sulphonylureas, the results showed a decreased risk of fracture; whereas the decreased risk of any fracture associated with the use of insulin was insignificant [10].

A multicenter study evaluated the effect of MF on 1259 Latin–American females aged over 40 years. They reported that using MF was associated with a lower risk of osteoporosis, regardless of the presence of type 2 diabetes or obesity [11]. 

Osteoblast production of osteoprotegerin (OPG) and receptor activator of nuclear factor kappa B ligand (RANKL) are both regulated by MF [12]. By decreasing osteoclast activity, the RANKL/OPG ratio stimulates bone growth and prevents bone resorption. The results of both surgical and nonsurgical periodontal therapy have improved as a result of the use of MF as an adjuvant treatment [13]. Araujo et al. in 2017 described the involvement of the Adenosine monophosphate-activated protein kinase/nuclear factor-κB (AMPK/NF-κB p65) and high mobility group (HMGB1) in periodontitis disease, as well as the effect of MF on oxidative stress, inflammation, and bone loss in a rat model [14]. The use of a topical application of metformin gel of different concentrations (0.5–1%) for the treatment of periodontitis has been reported in multiple clinical studies [15,16]. Bak et al. [9] conducted a comparison histological study using systemic MF in the management of ligature-induced periodontitis in rats. They found that the test group had a significantly lower rate of alveolar bone reduction compared to the control (vehicle-treated) group. It has been concluded that using MF has an influence on wound healing and, therefore, plays a significant role in implant survival rate due to the prevention of the undesirable effects of advanced glycation end products (AGEs) on osteoblastic cells, including interactions with receptors of advanced glycation end products (RAGEs) [17].

Pradeep et al. (2013) [15], in a randomized controlled clinical trial, utilized 0.5%, 1%, and 1.5% of MF as an adjunct treatment of intrabony defects in periodontitis compared with scaling and root-planing procedure (SRP). There was a significant reduction in intra-bony defect depth in the MF groups compared to the placebo group, with the greatest reduction in the 1% MF group [15]. Another study, published by the same author in 2015, compared the efficacy of open-flap debridement (OFD) combined with platelet-rich fibrin (PRF), 1% MF gel, and PRF + 1% MF gel in the treatment of intrabony defects (IBDs) in patients with chronic periodontitis. PRF, 1% MF, and PRF + 1% MF groups showed significant pocket depth (PD) reduction and relative attachment level (RAL) gain compared to the open-flap debridement (OFD)-only [18]. Mean PD reduction and mean relative attachment level (RAL) gain were noticed to be greater in the PRF + 1% MF group compared to just PRF or MF at 9 months [15,16,18].

The impact of topical MF as an adjunctive treatment was also evaluated with SRP in comparison to a placebo gel. The mean reduction in pocket depth (PD), gain in clinical attachment level (CAL), and intrabony defect depth (IBD) reduction were greater in the MF group at all time points [15]. The same effect was investigated in a randomized clinical trial, which used a controlled-release gel of MF adjunctive to SRP in the treatment of vertical defects in smokers with generalized chronic periodontitis [19]. It was concluded that there was more improvement in periodontal parameters in terms of vertical fill in SRP-treated sites with locally delivered MF, compared to sites treated with SRP with placebo only [19].

In an animal study on rats, it was reported that MF administration improved peri-implant bone healing in type 2 diabetics by the lowering of blood glucose levels. Rats that have controlled blood glucose levels by MF have remodeling biomarkers that are similar to the control animals [20]. Although the use of MF in the management of a variety of periodontal conditions has been reported in clinical and preclinical studies, the literature on its effect on peri-implant tissues is scarce [21,22,23,24,25,26,27]. Few studies have reported beneficial or equal effects as those of other standard, nonsurgical periodontal treatments [23,27]. Thus, the purpose of this review paper is to observe the impact of MF on the periodontal and peri-implant hard- and soft-tissue healing.

## 2. Materials and Methods

### 2.1. PICO Questions

The investigation question was “does MF have a positive effect on the healing of periodontal and peri-implant soft and hard tissues?”

### 2.2. Selection Criteria

The inclusion criteria of this review: (1) research papers published only in the English language, (2) animal studies, (3) clinical studies, and (4) studies on the systemic use of MF in periodontal and peri-implant soft- and hard-tissue healing. The exclusion criteria: letters to the editor, culture studies, and studies without controls.

### 2.3. Search Methodology

The keywords used in the electronic search were ‘metformin’, ‘diabetes’, ‘periodontitis’, ‘implant’, and ‘peri-implantitis’ via the Medline (PubMed) and Google Scholar databases for relevant articles.

### 2.4. Clinical Significance

To ascertain and confirm the exact clinical benefit of MF in terms of improved periodontal health, especially on bone regeneration, since there is still scarce information on in vivo and in vitro investigations on the impact of MF on periodontal and peri-implant soft- and hard-tissue healing following nonsurgical periodontal treatment and periodontal surgeries among diabetic patients.

## 3. Results

### 3.1. Impact of Metformin on Periodontal Health

**Figure 1:**
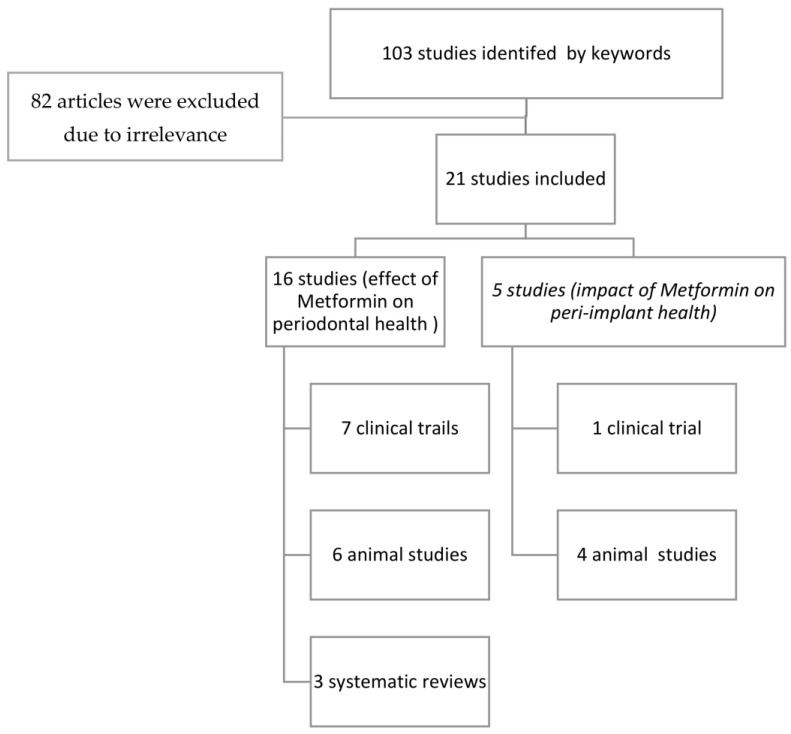
Flowchart of the included studies.

An electronic search using the keywords yielded 103 articles. The full papers of nineteen selected articles were strictly scrutinized for relevance following the inclusion criteria by authors. If there was any difference in opinion on inclusion or exclusion, it was resolved by taking the opinion of the third author, and then the articles were finalized. A final total of nineteen articles were included in the review (Figure 1).

Out of the studies shown in Table 1, six of the articles were animal studies [9,27,28,29,30]. In addition, seven clinical trials [15,16,18,19,31,32,33] are described in Table 2. Additionally, three systematic reviews [13,34,35] were included and are described in Table 3.

#### 3.1.1. Animal Studies

The in vivo animal studies tested the systemic and topical use of MF in rats. Their follow-up periods ranged from 10 to 84 days [9,14,28,29,30]. All the studies have reported a positive impact of MF on decreasing bone loss, oxidative stress, and process of inflammation. Studies have reported a positive effect on alveolar bone regeneration in periodontitis. It also has shown improved delayed gingival wound healing. One of the studies reported a favorable effect on alveolar bone in periodontitis by elevating the osteoblast differentiation [9] (Table 1).

#### 3.1.2. Clinical Studies

Six of the included clinical studies tested the adjunctive use of topical application of MF in IBDs [15,16,19,31,32,33]. One was in a smoker population [18]. All the studies used clinical and radiographic parameters, and the follow-up time ranged from 6 to 12 months. One study described the use of topical MF for moderate–severe chronic periodontitis management [18]. All the included papers reported a positive effect of MF compared to control through the clinical and radiographic evaluations [15,16,18,19,31,32,33].

#### 3.1.3. Systematic Reviews

Three systematic reviews and a meta-analysis were included [13,34,35]. Two of the reviews were on randomized controlled trials (RCTs) only [34,35], and one included two animal studies [13]. Although heterogeneity is evident between the studies, the use of MF shows a positive effect on periodontal health.

### 3.2. Impact of Metformin on Peri-Implant Health

#### 3.2.1. Animal Studies

A total of four animal studies evaluated wound healing and peri-implant tissue response (osseointegration) (Table 4). Three of them reported a positive impact of MF on healing of bone around the implant [20,23,24]. However, one study failed to prove the function of MF in altering the adverse effects of hyperglycemia, and the last study found in nondiabetic rats that MF had a negative effect on osteointegration [36].

#### 3.2.2. Clinical Studies

A retrospective study assessing clinical parameters around implants in patients using different hypoglycemic medication demonstrated that, depending on the hypoglycemic drug, bone remodeling around implants may vary [26]. Compared with classic hypoglycemic medication, the positive role of glucagon-like peptide 1 (GLP-1) drugs did not vary in bone remodeling around implants.

## 4. Discussion

The present review paper intended to study the impact of MF on periodontal and peri-implant soft- and hard-tissue healing among diabetic patients.

Due to microvascular changes, diabetics are at greater risk of developing postoperative infections, socket dimension alterations after extraction, and impaired wound healing in comparison to the nondiabetics; thus, DM may have negative impacts on the peri-implant hard and soft tissues [37]. An animal study conducted in 1996 [38], which compared uncontrolled diabetic rats with controlled and healthy ones, demonstrated that ten days post-extraction, thick collagen fibers formed a pretrabecular scaffold in the healthy and controlled diabetic rats, which influenced the direction of trabeculae formation. In contrast, the diabetic socket had collagen fibers that formed a narrow layer in the apical part as they were thin and insufficient. There was no evidence of diabetic microangiopathy in the extraction sockets of diabetic, insulin-treated diabetic, or normal rats [37,38,39,40]. Nevins and his colleagues reported that high serum glucose levels affect bone healing as a consequence of irreversible binding of glucose to the amino groups responsible for forming advanced glycation end products (AGEs) [39].

MF is one of the most prescribed oral antihyperglycemic drugs treating type 2 diabetes mellitus [41,42]. According to the Canadian Diabetes Association (2008), MF can also be used for weight loss in obese patients [43]. Furthermore, MF also showed a protective association in terms of bone loss in diabetic patients [28,29]. In addition, MF positively impacts the periodontium by increasing osteogenic gene expression and leading to osteoblast differentiation, thereby reducing bone loss [37,40,42,44]. Furthermore, MF reduces the oxidative stress and inflammation process and improves periodontal wound healing in both in vivo and in vitro investigations.

It has been known that there is an increased risk of bone fractures and impaired bone healing in patients experiencing uncontrolled diabetes [45]. To exclude the fact that metformin by itself has a significant positive impact on increased bone mineralization in individuals with controlled diabetes, we must look at the effects of other antidiabetic drugs on bone mineralization. For example, thiazolidinedione, an insulin-sensitizing medication, has a detrimental effect on bone mineral density by increasing osteoclastic activity [42]. On the other hand, metformin has the opposite effect, increasing bone mineralization by inhibiting osteoclastic activity via activating the AMPK signaling pathway, which, in turn, increases the proliferative action of osteoblastic activity that causes bone regeneration via mesenchymal stem cell differentiation [42,46,47]. At the same time, metformin controls glucose levels by inhibiting gluconeogenesis [48]. In the assumption of bone healing and regeneration, which is due to controlled diabetes through the use of antidiabetic drugs, thiazolidinedione would have not shown its deteriorating effect on the bone-regeneration mechanism.

The results of the randomized clinical studies included in this review (seven studies) [15,16,18,19,31,32,33], which investigated the impact of metformin on IBD and moderate-to-severe chronic periodontitis, have reported the positive effect of MF on clinical and radiographic parameters compared to control. A greater decrease in PD and CAL gain were reported with significant IBD depth reduction with the adjunct use of MF. A similar conclusion was reported from the systematic reviews included in this review [13,34,35], indicating that there is significant bone defect fill, reduction in probing depth, and gaining of the CAL with adjunctive locally delivered MF compared to control. However, the majority of the included studies were of short duration. The range of follow-up period was 3–9 months, and the outcome reported based on short-term periods was debatable. To ascertain the exact clinical benefit, long-term clinical investigations are required to appropriately evaluate MF’s effect on periodontal health.

It needs to be understood that the main outcomes of these reviews included assessments of the clinical periodontal parameters only, whereas no studies about the impact of the MF on wound healing and peri-implant tissue response were found.

On the other hand, reported results in the literature regarding peri-implant health seemed to suggest that MF may aid in osseointegration in diabetic patients. However, these results are to be viewed with caution since only one retrospective clinical study [26] and four animal studies were found [20,23,24,36]. Two of the Four animal studies reported a positive effect of MF on bone healing around implants [20,24]. One study introduced metformin after 15 days of implant placement, while another study looked at the effect of MF on nondiabetic rats. Both, however, did not report a positive effect [23]. Furthermore, the clinical study comparing three different antiglycemic drugs did not find a positive effect of MF on the bone around implants based on clinical and radiographic parameters. This result could be due to study design and other confounding factors [26]. Future RCTs should be conducted to better assess MF impact on bone healing around implants. It is also to be noted that two animal studies showed a negative impact on bone repair [30,36], which negatively affected osseointegration by lowering the percentages of BIC and BA and increasing the expression of RANKL introduced in nondiabetic rats [36]. However, both studies were not conducted on a diabetic animal, which may explain this outcome. Despite the reported positive impact of using MF as an adjunctive agent on periodontal health, studies that have assessed peri-implant soft and hard tissues are limited. Moreover, there are no studies that have compared the healing of implant-related procedures (alveolar ridge preservation and guided bone regeneration) in a diabetic patient using MF with other antidiabetic agents. Besides that, no identification of age groups has been considered in the previous studies. Thus, additional studies are needed before implementing the use of MF in nondiabetics to clearly confirm the impact of MF on diabetic patient bone health. Due to limited studies including clinical and animal experiments, it is recommended to conduct further clinical and animal trials to reach a more clear insight of the effect of metformin and its impact on hard- and soft-tissue healing.

## 5. Conclusions

Although there is lack of evidence showing the effect of MF on periodontal and peri-implant health, some animal and clinical studies have demonstrated a positive/protective impact of MF. Future cohort studies evaluating this relationship longitudinally may aid in understanding the temporal sequence and dose response impacts of MF.

## Figures and Tables

**Table 1:** Animal Studies on the impact of Metformin on periodontal health.

Study (Author and Year)	Study Design	Types of Periodontal Lesions	Methods	Intervention	No. of Subjects	Follow -Up	Outcomes
Pereira et al., 2018 [28]	In vivo prospective	Ligature-induced periodontitis	Histopathological, immunohistochemical analysis and Micro-CT analysis	MET-loaded PLGA	Control 80 Test: 80	10 days	MET-loaded PLGA reduced inflammation and periodontitis-associated bone in diabetic rats.
Xu et al., 2021 [29]	In vivo prospective	Ligature-induced periodontitis	Micro-CT and histological analysis	Composite scaffold loading metformin (MET) implanted in the alveolar bone defects	Not reported	7 and 14 days	MET has a positive impact on alveolar bone regeneration in periodontitis.
Araújo et al., 2017 [14]	In vivo prospective	Ligature-induced periodontists	Histopathological, immunohistochemical analysis and micro-CT analysis	MET-loaded PLGA	Five groups composed of twenty-one rats each: (1) Without ligature and water. (2) With Ligature and water. (3) Ligature with 50 mg/kg MF. (4) Ligature + 100 mg/kg MF. (5) Ligature + 200 mg/kg MF.	11 days	Metformin reduces the inflammation, oxidative stress, as well as bone loss.
Kominato et al., 2022 [30]	In vivo prospective	ligature-induced periodontist	Histological and histomorphometry analysis, in vitro wound healing, and cell proliferation assay	Metformin effects and the underlying mechanism in the acceleration of gingival wound healing under minimal influence of glycemic control	20 mice	7 days	Metformin enhanced delayed gingival wound healing in insulin- resistant prediabetes.
Malta et al., 2020 [27]	In vivo prospective	ligature-induced periodontitis	Histometric and immunohistochemical analyses	Oral metformin	80 rats	84 days	Metformin and hyperglycemia have negative influence on bone repair.
Bak et al., 2010 [9]	In vivo	Ligature-induced periodontitis	Histologic examination and microcomputed tomography	Oral metformin	Control: 5 Test: 5	10 days	Metformin increases the osteoblastic differentiation in periodontitis, which may exert a favorable impact on alveolar bone.

**Table 2:** Clinical studies of Metformin impact on periodontal health.

Study (Author and Year)	Study Design	Types of Periodontal Lesions	Methods	Intervention	No. of Subjects	Follow -Up	Outcomes
Rao et al., 2013 [19]	RCT	Vertical bony defects in smokers with generalized chronic periodontitis	Clinical and radiographic parameters	SRP plus 1% MF (local delivery) (35 sites)	SRP plus placebo (36 sites)	6 months	Pocket reduction and mean values of CAL gain > in the MF group at all of the follow-up visits. MF group shows significantly higher mean percentage of bone fill (26.17–6.66%) vs. placebo sites (3.75–8.06%)
Pradeep et al., 2013 [15]	RCT	Intrabody defects (IBDs) in patients with chronic periodontitis.	Clinical and radiographic parameters	(30 sites) SRP and placebo groups	(3 groups of 30 sites each) SRP with 0.5%, 1%, and 1.5% local MF gel.	6 months	At 3 and 6 months, mean values of PD reduction and CAL gain were more in MF groups than the placebo. Significantly greater reduction in the depth of IBD in the MF groups in comparison to the placebo group, with greatest decrease of 1% MF.
Pradeep et al., 2015 [16]	RCT	Intrabody defects (IBDs) in patients with chronic periodontitis (CP).	Clinical and radiographic parameters	OFD alone (32 sites)	32 sites were treated with OFD and PRF, 31 sites treated with OFD with 1% MF, and 31 sites with OFD and PRF plus 1% MF	9 months	Significantly greater reduction in PD and RAL gain in the groups of PRF, 1% MF, and PRF + 1% MF than the OFD-only group. PRF + 1% MF group had greater reduction in PD and mean RAL gain and significantly higher percentage of radiographic defect depth reduction compared to PRF or MF alone at 9 months.
Kurian, et al., 2018 [31]	RCT	Intrabody defects in chronic periodontitis patients.	Clinical and radiographic parameters	SRP + placebo gel (30 sites).	30 sites: SRP + 1% MtF gel) 30 sites: SRP + Aloe veragel).	12 months	Significant PPD reduction and CAL gain at 6 and 12 months of using 1% MtF group compared to the AV and placebo groups.
Pradeep et al., 2016 [18]	RCT	Moderate and severe chronic periodontitis (CP).	Clinical and radiographic parameters	SRP plus placebo (34 sites)	SRP plus 1% MF (36 sites)	9 months	In CP patient sites treated with SPR and locally delivered, MF had greater decrease in PD and more CAL gain with significant intrabony defect depth reduction
Pankaj et al., 2018 [32]	RCT	Intrabody defects in chronic periodontitis	Clinical and radiographic parameters	(30 sites) received SRP plus placebo gel	30 sites: SRP plus 1.2% RSV gel 30 sites: SRP plus 1% MF gel	12 months	Significant PD reduction and CAL gains, as well as improved bone fill reported with locally delivered 1.2% RSV and 1% MF gel in comparison with placebo gel.
Khalifehzedeh et al., 2019 [33]	RCT	Two-wall intrabody periodontal defects.	Clinical and radiographic parameters	OFD (6 sites)	6 sites each: 1% MF, plasma rich in growth factor (PRGF), and PRGF and MF.	6 months	The group of 1% MF with PRGF showed significant radiographic changes when compared with other groups.

**Table 3:** Systematic Reviews on the impact of Metformin on periodontal health.

Author	Intervention and Defect Studied	Number and Types of Included Studies	Outcome
Akram et al., 2018 [34]	Adjunctive use of locally delivered MF to SRP in treatment of periodontal defects	5 RCT and 3 in meta-analysis.	All the involved experiments revealed significant BD fill, PD reduction, and CAL gain with adjunctive use of locally delivered MF as opposed to SRP alone. Meta-analysis: a statistically significant PD reduction (WMD = −3.11, 95% CI = −3.63 to −2.59, *p* < 0.001) and CAL gain (WMD = −2.83, 95% CI = −3.32 to −2.34, *p* < 0.001), BD fill (WMD = −2.96, 95% CI = −3.99 to −1.93, *p* < 0.001) for SRP + MF treatment compared to SRP.
Nicolini et al., 2019 [35]	Adjuvant effects of MF on the outcomes of mechanical periodontal treatment	4 RCT	For PD and CAL, the findings demonstrated a weighted mean difference of 2.12 mm (95% CI 1.83–2.42) and 2.29 mm (95% CI 1.72–2.86), respectively, favoring the group subjected to 1% adjunct MF.
Najeeb et al., 2018 [13]	MF in the treatment of periodontitis	2 animal studies 4 RCT	The topical usage of MF led to histological, clinical, and radiographic outcome improvements. Meta-analysis showed that application of MF improved the clinical and radiographic outcomes of SRP; however, a heterogeneity of the results was evident.

**Table 4:** Animal studies on the Impact of Metformin on peri-implant health.

Study (Author and Year)	Study Design	Methods	Intervention	No. of Subjects	Follow-Up	Outcomes
Bastos et al., 2017 [36]	In vivo prospective implant placement	Histometric measurements of bone-to-implant contact and bone area, in addition to immunohistochemical analysis	Metformin (40 mg/kg/day by gavage).	Control: 10 Test: 10	30 days	MF negatively affected osseointegration by decreasing both the bone area (BA) and bone-to-implant contact (BIC) percentages and increasing the expression of RANKL around titanium implants in nondiabetic rats.
Inouye et al., 2014 [20]	In vivo prospective implant placement	Microcomputed tomography and blood analysis	1 × 3 mm titanium in healed sites. Metformin (40 mg/kg).	Nondiabetics: 12 Diabetic: 12 Diabetic on metformin: 12	4 weeks	After metformin use, hyperglycemic type 2 diabetic rats presented improvements in blood glucose as well as wound healing around implants.
Yıldırım et al., 2020 [24]	In vivo (bone filling around implants in rats)	Histopathologic analysis	TiAl6Va4 implants inserted in the metaphyseal part of the tibial bone. Metformin (40 mg/kg).	Controls: 10 Metformin 10	28 days	The ratios of bone filling among the rats in the metformin group were 55.50 ± 14.034, and in the CNT group were 37.78 ± 13.017 (statistically higher).
Serrão et al., 2017 [23]	In vivo (reversing the negative effects of hyperglycemia in rats)	Histometric measurements: bone-to-implant contact (BIC), bone area (BA), κB ligand RANKL- and OPG-stained cells	Titanium implants were placed in tibiae. Metformin (40 mg/kg).	DM II DM II on metformin (40 mg/kg/day), starting on the 15th day after implant placement). Control group: nondiabetic rats without MF treatment.	30 days	The percentages of BIC and BA in the cortical bone were decreased in the DM and MDM groups opposing the control group (*p* < 0.05) in the DM group compared with the control group. The percentage of BA in the medullary region was decreased (*p* < 0.05). The highest number of OPG-stained cells was in the MDM group; however, the highest ratio of RANKL/OPG in the medullary area was in the DM group (*p* < 0.05).

## Data Availability

Not applicable.
